# J. W. W. Gordon’s Vaccinator

**Published:** 1864-04

**Authors:** 


J. W. W. GORDON'S VACCINATOR.—We have received this instrument from the inventor and manufacturer, and among all the mechanical appliances recently invented for the purposes of facilitating the operations of the physician, there is, perhaps, not one which surpasses in value and convenience this instrument. In the plan of construction it resembles a spring lancet, a small capillary tube being substituted for the lancet. This point is excavated for the reception of the virus, and is charged by simply dippng it in lymph or dissolved scale. The case contains vaccinator, plates of glass for rubbing up the vaccine scale or for lymph, and a bottle suitable for preserving virus. Physicians who desire to make this operation frequently, or who would do it so that children have neither occasion or time to cry, cannot do better than to send for this instrument. We have ourselves been very greatly pleased with its operation, and think it superior to any vaccinator heretofore invented.

